# A Heterogeneous IoT Data Analysis Framework with Collaboration of Edge-Cloud Computing: Focusing on Indoor PM10 and PM2.5 Status Prediction

**DOI:** 10.3390/s19143038

**Published:** 2019-07-10

**Authors:** Jaewon Moon, Seungwoo Kum, Sangwon Lee

**Affiliations:** 1 Information & Media Research Center, Korea Electronics Technology Institute, Seoul 03924, Korea; 2 Department of Interaction Science, Sungkyunkwan University, Seoul 03063, Korea

**Keywords:** decentralized analysis architecture, edge computing, heterogeneous IoT data analysis, collaborative analysis

## Abstract

The edge platform has evolved to become a part of a distributed computing environment. While typical edges do not have enough processing power to train machine learning models in real time, it is common to generate models in the cloud for use on the edge. The pattern of heterogeneous Internet of Things (IoT) data is dependent on individual circumstances. It is not easy to guarantee prediction performance when a monolithic model is used without considering the spatial characteristics of the space generating those data. In this paper, we propose a collaborative framework using a new method to select the best model for the edge from candidate models of cloud based on sample data correlation. This method lets the edge use the most suitable model without any training tasks on the edge side, and it also minimizes privacy issues. We apply the proposed method to predict future fine particulate matter concentration in an individual space. The results suggest that our method can provide better performance than the previous method.

## 1. Introduction

In recent years, big data analytics have gained increased attention to analyze data based on the Internet of Things (IoT). IoT data analytics require technologies that can transform a large amount of data into more understandable information to discover meaningful patterns and trends. Various IoT devices and sensors are deployed to provide convenient services with machine learning models. Presently, some cloud platforms provide machine learning services with pre-trained models to understand IoT data easily [1]. When it comes to functional aspects, the cloud is the best solution to handle big data due to its scalability, cost-effectiveness, and usability.

In some situations, where fast response is the most important variable, it is not efficient to process all of the data in the cloud. This is because the cloud might be far from the location of sensors and devices generating time-series data. in this scenario, the analyzer in the cloud cannot process data in real time. It also takes more time to transfer the results to a local actuator when data are processed near the edge. Moreover, delivering personal data to the cloud might cause privacy concerns. It is inevitable that there would be privacy concerns due to local data transfer. In addition, unexpected network problems might prevent a customer from getting analysis results within an appropriate period of time. 

The edge platform [2,3,4] is a good alternative to overcome the disadvantages of using machine learning models in the cloud platform. The edge platform is an optimized cloud computing system that performs data processing at the edge of the network and allows computing resources to reside close to the end device, thereby reducing communication latency. Data can also be analyzed within a single hop distance from the end device. Common devices, such as routers, switches, and video surveillance cameras, can be used as an edge node if they have computing, storage, and network connectivity capabilities. To overcome the aforementioned problems, some specific analysis tasks have been moved to the edge platform. Many IoT applications rely on efficient, real-time processing. Recent advances in IoT data via neural networks have enabled new applications that can improve the quality of the service. Analyzing IoT sensor data in the cloud results in latency due to data movement and processing, and services are network dependent [5,6]. Alternatively, processing IoT data at the edge is more suitable for real-time processing.

One of the biggest disadvantages of the edge platform is that it has relatively low data processing power. A challenge that faces these applications to edge devices is the requirement of real-time processing with low computation power; this is exacerbated by the fact that machine learning models are becoming increasingly complex. Most edge devices do not have enough capacity to process and train a large amount of IoT data [7]. Therefore, they generally use a model where the cloud platform sends in one-way. Moreover, since any device with processing capability can be the edge, it is not easy to give an absolute quality of service (QoS) guarantee. Various edges have different performance and there is no standard edge specification [8]. In addition, the capacity of normal edge storage is not enough to store big data, and the edge can only access and process data from nearby devices in a specific area.

Collaborative analysis between the edge and the cloud has the potential to avoid the disadvantages of both platforms. Recently, several studies related to distributed learning and analysis using both cloud and edge platforms have been introduced [9]. The most common method to distribute the task load is to generate general models in the cloud platform, transfer those models to the edge platform, and make inferences with the edge platform using transferred models [10].

Many countries are suffering from elements of air pollution, such as particulate matter (PM), with rapid industrialization. People usually spend more time indoors than outdoors, and the indoor air is more severely polluted than the outdoor air, which can be naturally purified [11]. Therefore, it is helpful to provide the prediction information of indoor environmental status promptly in order to understand trends of air change and to manage indoor air quality. However, IoT sensor data that are heavily dependent on the temporal status and variables related to the spatial characteristics of the space being measured should be considered differently from other homogeneous inputs, such as image and audio. For example, when considering the prediction of the future state of fine indoor dust, the characteristics of the space to be predicted and changes in the indoor air pattern with time will be very different in each space. In this case, when choosing a general model from the cloud platform and delivering it to all edges in a batch, it can be difficult to ensure the predictive performance because of the varying magnitudes of the effects of several factors on a specific factor.

In this paper, we propose a new method that can analyze IoT data by distributing roles. The proposed framework is designed to use the resources of the cloud to generate the analysis model and then select and transfer the best model for an individual edge. In addition, we propose a new method to select the most proper prediction model. To verify this framework, we considered a real use case that predicts the future concentration status of fine indoor dust. We collected actual data in several indoor spaces to verify this framework.

The remainder of this paper is structured as follows. Section 2 provides detailed background information, and the proposed framework architecture is introduced in Section 3. Section 4 explains the use case and related machine learning methodology, and Section 5 details the results based on an actual use case. Section 6 discusses the use case experiment results and presents the concluding remarks.

## 2. Related Work

Over the past few decades, many information technology (IT) companies have attempted to manage resources centrally in terms of infrastructure. Conducting analysis in the cloud has become a universal service with the development of analytical technologies. At the same time, distributed analysis has also been considered to reduce privacy concerns, network dependency, and latency arising during the processing of large amounts of data. Edge computing and cloud computing solutions have their own distinct advantages and disadvantages in terms of IoT data analysis and providing real-time service.

Table 1 describes the advantages and disadvantages of the cloud and edge platforms. In summary, the cloud has no restrictions on the use of storage and very high processing performance. Users only pay the service provider a cost based on the amount of use for resources. Since the network processes a large amount of data during transmission, cloud-based service is highly dependent on the status of the network; accordingly, it requires network bandwidth guarantees. Additionally, transferring user-side data to external cloud storage is inevitable, so an external network must be used to handle local data. For the aforementioned reasons, it is not easy to guarantee the quality of service (QoS) of real-time IoT analysis services [12]. This can cause a delay between a client request and a cloud service provider’s response. In addition, since data are transmitted to the outside, it is difficult to avoid privacy-related problems [13]. In contrast, the edge can process real-time data without connecting to external services because it is close to real sensors. Furthermore, it is not restricted by connection failures with an external network. For this reason, the edge platform can guarantee a better QoS. Considering the characteristics of each platform mentioned above, integrating the cloud’s centralized capabilities with the real-time benefits of edge computing can address a variety of challenges in terms of handling real-time data analysis during wireless IoT services.

Several types of studies related to edge-cloud collaborative analysis have been proposed over the last few years. In the case of cloud-centric analysis, all IoT-generated data are transferred to the cloud, and the cloud stores and uses data for training and inference. In this case, there is no need to consider the role of the edge except for data generation.

Alternatively, the cloud can generate an analytics model and the edge can use it for inference. This method helps the cloud and the edge collaborate more actively than the previous method. When the cloud delivers the trained model to the edge, the edge uses the delivered model to make predictions and inferences. Several major cloud service providers provide or are preparing to provide similar services. Greengrass [14] of AWS, which provides large-scale cloud services, is a representative service. Greengrass allows local computing, messaging, syncing, data caching, and machine learning inference for connected devices. However, this service can be strongly dependent on its legacy cloud platform. In addition, it is also not easy to obtain good results in a different environment because it usually provides a single generic model in the cloud [15,16].

Cloud and edge platforms can collaborate more actively for training and prediction. Several kinds of studies were introduced to improve the prediction performance individually based on federated learning using real-time generated data on the edge side [17,18,19]. In this case, even if real-time data are not sent to the cloud, the model can be upgraded continuously at the edge. However, previously proposed methods cannot be used for realistic collaboration between the edge and the cloud, with the exception of a few simple scenarios, such as message recommendation. Google [20,21] announced a study looking at how the edge could train itself. The proposed method can be applied to cases where the data size is small, such as keyboard input messages.

Studies that investigated the collaboration of analytical models using IoT data were first introduced in the field of image processing, and several open image datasets have helped image machine learning researchers advance related technologies. To address the shortcoming of image processing in either the cloud or the edge, research has used a distributed computing approach. Hierarchically distributed computing architectures have many advantages, such as providing coordinated central and local decisions and scaling intelligent tasks on geographically distributed IoT devices [2]. In addition, several distributed learning methods have been proposed to increase the computational efficiency and reduce the processing power by considering a neural network model using video sensor input. Various conventional methods have been combined, such as splitting prediction models separately and deducing each of them individually, transmitting compressed data from the edge to the cloud to determine the final result, and using the optimal prediction model from the cloud with adaptation. 

Training a machine learning model requires a relatively high performance due to repeated computing a large amount of data. Training cannot be performed on an edge where performance is not sufficient. However, an inference could be fine on edge with relatively low performance since it does not require all the infrastructure of training. Therefore, depending on the service scenario, latency can be reduced getting the inference result computed on the edge directly without communicating with the cloud. In addition, edge inference can reduce the dependency on network connections and improve user experience. [22].

Some studies have also shown that the edge can extract important features from input data, which can be used to derive the final result on the cloud side. For this method, a single learning model is needed; this is separated, and each model is placed in the cloud and the edge, respectively [23,24,25]. Kang et al. [9] proposed a method to divide the neural network into edge and cloud partitions. In this paper, the neural network was divided into an edge side and a cloud side. This helps optimize the bandwidth between the edge and the cloud, reduces processing latency, and leads to lower energy consumption. When the cloud and the edge are tightly coupled together, such as in splitting prediction models, the workload can be distributed; however, the dependency on the cloud remains. Private information can be removed by edge processing, and only partial feature data are transferred to the cloud by this method. Here, the cloud derives the result by a final inference. However, a certain amount of partial data needs to be sent to the cloud, so this may not be a good solution to solve network bandwidth dependency concerns. It can also be applied to only a few models that have a special structure that can be easily divided, such as the convolutional neural network (CNN). In addition, this does not solve the delay problem because only the cloud can produce the final result. Because this approach is cloud-dependent, it makes independent service execution difficult. In conclusion, this is not a general way to obtain a good result in various domains. 

Kang et al. [26] proposed a method to compress data from the edge before it is sent to the cloud. In this case, the edge sends compressed data to the cloud to reduce computational overhead. However, the prediction accuracy becomes lower due to compression. When compressed data are sent from the edge to the cloud, the original local data may be lost and the performance might be degraded. Another method is sending the frame difference between the old frame and the next one [27,28]. To reduce communication bandwidth, the edge only sends the difference compared to the previous frame to the cloud, as opposed to sending the whole frame. In general, these methods aim to minimize the amount of transmission between the edge and the cloud by considering the capacity and processing speed of the video data and distributing the work in two separate ways. However, determining the right parameters is difficult. To address this disadvantage, a previous study attempted to integrate these methods to achieve higher performance [29]. Another study investigated edge-cloud collaborating processing to improve the quality of DNN processing at the cloud by providing feedback to the edge. This study proposed a method to transfer useful information of IoT devices to the end task with the highest quality [30].

Previous works have mainly proposed collaboration technologies for homogeneous data. Most of the aforementioned methods are suitable for only homogeneous data, such as image, audio, and text. However, these methods cannot be applied to heterogeneous IoT sensor data sets. In the case of image data, it is reasonable to use the best forecasting model created in the cloud without modification. However, this is not suitable for time series heterogeneous data sets. Most values of IoT sensors are affected by time and space, as well as many other variables, on an irregular basis.

**Figure 1:**
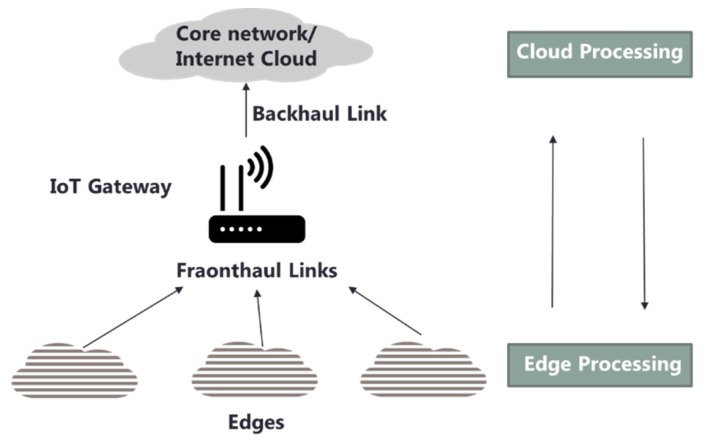
Generalized system model of collaborative edge-cloud processing.

Many studies have recently been introduced to use and analyze heterogeneous IoT sensor data on the edge. The efficiency of machine learning for IoT has been evaluated in many IoT application areas such as wearable sensor data analysis [31], deep learning method at edge [26,32], and architecture considering edge network [33]. However, there are few studies about heterogeneous IoT sensor data analysis that consider collaboration and decentralization. Only a few recent studies have been introduced that suggest abstracted methodologies related to this topic. Figure 1 shows the generalized system model for collaborative edge-cloud processing. The heterogeneous raw data coming from different sensors and spaces are diverse and continuously collected over time. 

In a previous study [34,35] potential key enablers and the associated key challenges for collaborative edge-cloud frameworks were presented. This study mentioned eight potential enablers and challenges: coordination mechanisms between edge computing and cloud computing, big-data-aware edge-cloud collaborative processing, task/data/computation/program offloading, adaptive optimization of computing, communications and caching resources, autonomous device collaborations in wireless IoT networks, data-driven event monitoring and event-driven service provisioning in wireless IoT networks, software-defined networking and virtualization in wireless IoT networks, and security/privacy enhancement in wireless IoT networks. This paper also mentioned the importance of adaptive learning and prediction algorithms for collaborative learning. It proposed future directions of collaborative frameworks focusing on improving the efficiency of the computing ability when considering the structural aspect of wireless communication between the edge and the cloud. However, it deals only with the overall challenges and needs of a collaborative framework; it does not provide a specific use case or detailed algorithm method.

In another paper [36,37], the theoretical methods of adaptation, learning, and optimization in multi-agents on the network were presented. This paper dealt with the topic of information processing over graphs and how collaboration among agents in a network can lead to superior adaptation and learning performance. It considered three main topics: how to perform distributed optimization over networks, how to perform distributed adaptation over networks, and how to perform distributed learning over networks.

However, it is not easy to find use cases that handle real, heterogeneous IoT data. The first reason that it is more difficult to conduct edge-cloud collaborative analysis studies on IoT data than on image data is related to difficulties in obtaining a heterogeneous IoT sensor data set. Additionally, there is not a standardized format to describe various IoT data sets. Moreover, a heterogeneous IoT data set depends on time, space, and other related factors. To analyze heterogeneous IoT data, it is necessary to account for the relationships between many factors. 

When predicting heterogeneous IoT data values, time and space dependency is especially high and affected by many other factors [38,39]. If the machine learning model from the cloud is used without optimization in the edge [40,41], it may be difficult to achieve the desired goal due to the lower performance of the edge. The method of analysis at the edge must be clearly distinguished from the method of analysis in the cloud, which can access high processing power and storage. Transfer learning [42,43] or fine-tuning has been used to improve the performance of the original model. The transfer leaning method is especially popular in image processing. However, not all analytical problems produce good results. 

PM [44] is a general term for extremely small particles and liquid droplets in the atmosphere. Primary sources come from incomplete combustion, automobile emissions, dust, and cooking. In addition, it is also known to be produced by chemical reactions in the atmosphere. In addition, exposure to particle pollution cause increases in a health hazard. When inhaled, particle pollution can travel deep into the lungs and cause heart and lung diseases. Therefore, we need to able to predict future indoor conditions of the particle contamination to properly cope with such environmental pollution and protects health [45].

To overcome the aforementioned challenges, we propose a framework that provides an optimal model with minimal data exchange between the edge and the cloud. The framework can solve problems related to private data being sent to the cloud and high network dependency. To verify the proposed framework, a test data set from an indoor environmental sensor was collected to predict the future fine dust status in an indoor space. 

## 3. Framework Based on Edge-Cloud Analysis Collaboration

In this section, we introduce a new framework for edge-cloud collaboration. The cloud and edge platforms work together to find the best analytical model. Training on the edge is still challenging due to cost, form factor limitations, latency, power consumption, and other considerations. On the other hand, a real-time inference can be performed on the edge device. Therefore, the edge needs to find the most proper machine learning model from the cloud and use it for quick inference. We considered a real use case to make a more concrete framework. 

### 3.1. Proposed Framework Architecture

The proposed framework is an efficient collaboration between the edge and the cloud to protect privacy and reduce network traffic. We considered the following requirements.

- The cloud has to help unload the computational burden of the edge and transfer the proper model by considering local data characteristics.
- The edge has to send as little data as possible to the cloud and be able to use the final results as quickly as possible.

**Figure 2:**
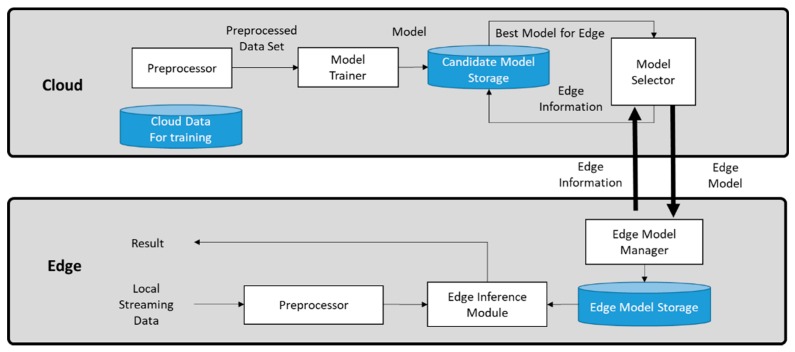
Proposed framework structure.

Figure 2 shows the proposed framework structure. This paper deals with generating predictive models by using inputs from various sensors collected in different spaces. The cloud collects and preprocesses data before training. Public data from many spaces have different ranges, frequencies, and formats. Therefore, it is essential to integrate data into the appropriate format and preprocess it for machine learning. The model trainer uses this preprocessed data to create different individual prediction models that account for the capabilities of the various edges and the individual IoT data characteristics. Then, the model selector chooses the best model and sends it to the edge along with the individual edge information.

The edge can handle local real time data and communicate selected information to the cloud. The cloud delivers the most appropriate model to the edge based on the information transferred from the edge. Then, the edge inference module uses the delivered model to predict and derive results. The edge model manager stores the delivered model from the cloud in the edge model storage. This allows the edge inference module to use the stored model to generate predictions quickly. The results generated by the edge inference module can be used for any service at the edge directly.

### 3.2. Model Selection

This section describes how to generate candidate models and select the appropriate model for use on the edge. The role of the model selector is to deliver the most proper model to the local edge according to its characteristics. The larger the data that the edge sends and receives to/from the cloud, the higher the network dependency and the greater the privacy-related concerns. In this paper, the edge transmits minimum sample data to select the optimal model. This means that only selected data of a certain period are sent instead of transmitting all of the data. Additionally, the prediction model can be chosen based on the correlation between the edge and the training dataset. 

To predict multiple factors in different spaces, individual pieces of data in each space must be used independently for learning. When 2 sensor values are predicted for M spaces, 2 × M prediction models are generated. The most suitable model is transferred to the edge according to the feedback of the ML model selector.

Its data type must also be converted to a format suitable for training input. For example, when long-short term memory (LSTM) is used as the main machine learning method for the test in this paper, data from a specific period (not data for a specific time point) are used as an input. In this paper, LSTM is used as a representative prediction algorithm to test the proposed framework; however, it should be noted that other prediction algorithms can be also used.

**Figure 3:**
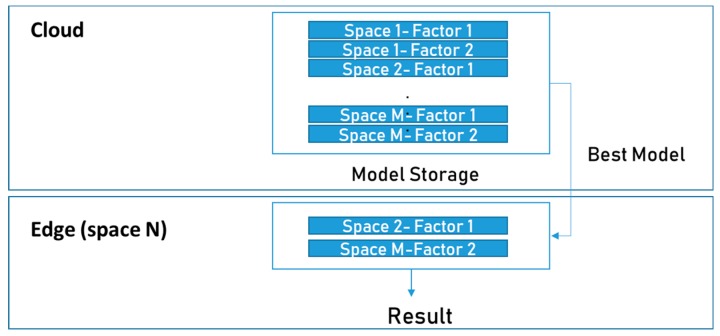
Simple model selection flow to find the most proper model.

Figure 3 shows the simple data flow used to find the right model at the edge. First, the cloud collects public data for training at each space and generates a model for each station that can predict various factors based on the data. At the edge, some of the locally collected data are transferred to the cloud for initial model selection. This solves the privacy problem by sending only the initial sample data, for which the user’s permission has been granted, but not all of the real-time data. The cloud computes the correlation between the local data of the factor to be predicted and the data of the factor of the public spaces used for training over time and determines the space with the highest correlation. Then, the prediction model generated based on the selected space is selected as the most suitable model; this is used in the edge. If the user does not want to send even the sample data, it is possible to take sample data from the cloud, calculate the correlation at the edge, and determine the appropriate model information at the edge. The edge model manager can deliver the least amount of information to the cloud. 


**Algorithm 1. Model Selection**
Information:  n = the number of sample space   f = the factor to be targeted  D = The set of sample data crawled from n sample spaces  d = The data set that was crawled from the new edge space to be predictedInput:   **D_f_** = partial data of D including only f factor  **d_f_** = partial data of d including only f factor  M_f_ = n candidate models predicting the future status of factor f based on individual sample space dataOutput:   Edge_modelfunction:  Model_selection(**D_f_**, **d_f_**, M_f_)  C = correlation(**D_f_**, **d_f_**)  Edge_model_num = C.index(max(C))  Edge_model = get_model(Mf, Edge_model_num)  return Edge_model

For example, the service provider generates models that predict the future status of factor1 and factor2 with public data from N spaces. Additionally, it wants to deploy the same prediction service for a new SpaceN. However, a new user staying at SpaceN does not want to transfer environmental information indiscriminately to the cloud. In addition, it is also assumed that there is no appropriate edge that can make the prediction model because it does not have sufficient computing resources by itself at SpaceN. In this case, the cloud cannot train local data to generate its own model and the edge does not have sufficient computing power for training; therefore, it is impossible to generate a predictive model. The only way to provide the prediction service for new users in SpaceN is to use a predefined model in the cloud. However, for time series IoT sensor data prediction, which is highly affected by several various complex factors, it is difficult to use a general standardized model because it depends on the spatial and temporal characteristics of each space. To overcome this problem, this paper proposes a new method. Only sample data of SpaceN are transmitted to the cloud as information for selecting the most suitable model. 

Algorithm 1 describes the detailed procedure of model selection. The model selector calculates the correlation Df and df where f is the factor to be targeted, D is the set of sample data from n sample spaces, and d is the data set from new edge space for the same time duration. Then, it finds the most similar space that has the highest correlation and sends the model trained by data from this space. For example, if factor1 data of Space2 have the highest correlation with SpaceN during the time interval of the sample data, the factor1 prediction model trained with Space2 data is transferred to the edge in SpaceN. Also, the factor2 prediction model of SpaceM can be used if the factor2 correlation between SpaceM and SpaceN is the highest.

## 4. Use case and Methodology

### 4.1. Use Case

Many IoT-based services are used for real-time indoor safety monitoring. However, the focuses of current services are mostly restricted to remote monitoring of the current situation with devices like smartphones. Moreover, these services are usually based on the cloud. The round-trip delay induced by the cloud may not be suitable for time-critical services. Moreover, privacy concerns may arise because data are uploaded to the public cloud.

We have considered the use case scenario of predicting current and future indoor conditions based on indoor and outdoor statuses. Outdoor air has a relatively similar effect on large areas. Therefore, we assumed that all outdoor conditions of each indoor space are the same. However, we have hypothesized that the outdoor condition has a different impact on each indoor environment. The degree of the influence of outdoor air on each indoor space is different because each indoor condition is very different and independent. Therefore, it is necessary to consider the different relationships between the indoor and outdoor environments for each space. For example, even if the concentration of outdoor PM is the same, the effect on indoor spaceA and spaceB will be different. Therefore, if this hypothesis is true, the prediction model for each environmental factor of each space should be created independently.

To specify the structure of the framework, we predict the future using multivariate time series variables. We have assumed a use case to improve the conditions of a specific space by using the proposed framework. This requires an inference engine that can predict the future status of a specific space based on historical data. Three types of predictive models for each space were created for the test, as follows:

- 1^st^ scenario: PM10 and PM2.5 status prediction after 1 h using the last 2 h of data
- 2^nd^ scenario: PM10 and PM2.5 status prediction after 20 min using the last 30 min of data
- 3^rd^ scenario: PM10 and PM2.5 status prediction after 25 min using the last 60 min of data

For example, in the first scenario, the models can predict the status of PM10 and PM2.5 after 1 h using indoor/outdoor data for the past 2 h. The goal of use case is to select and provide the most suitable model among N models already generated for the edge. The results of the inference engine should be profiled to quickly control the IoT actuator to improve the current status in a specific case. The cloud provides the edge with a model to predict the future status of fine dust. The cloud prepares and stores multiple candidate prediction models based on publicly available data sets that have already been selected for transmission and selects the best model for the edge. 

**Figure 4:**
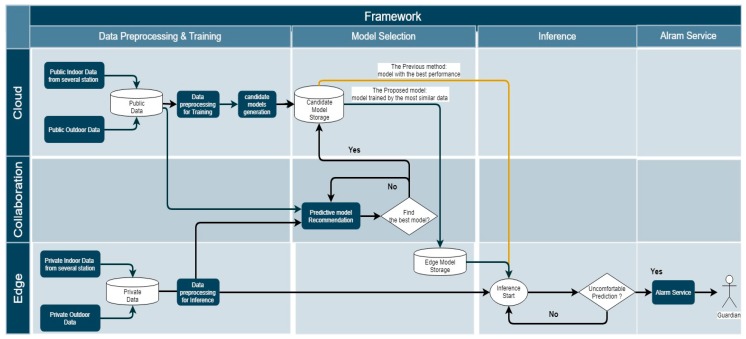
The dataflow of the use case.

Figure 4 shows the dataflow of the proposed framework considering the use case. The flowchart consists of four main procedures: data preprocessing and training, model selection, inference, and IoT interaction (alarm) service. The detailed functions are described below.

- **Data preprocessing and training (cloud):**
  ○ The cloud platform collects indoor and outdoor data from public stations and stores them in the database. Each set of space data for a public purpose is used for training after preprocessing.
  ○ Candidate predictive models are made and stored in the candidate model storage.
- **Data preprocessing (edge):**
  ○ The edge platform collects indoor data from a local space and stores them in the edge database.
  ○ A certain amount of historical data required for inference is preprocessed as an input.
- **Model selection:**
  ○ Selecting a predictive model requires local and cloud data.
  ○ In this paper, the model was selected by transmitting only local sample data to the cloud. However, if a user does not want any local data to be sent to the cloud, the cloud can send its data back to the edge.
  ○ The predictive model recommender chooses the most appropriate model based on data from both the cloud and the edge.
  ○ The selected model is stored in the edge model storage.
  ○ The edge can use the model of the edge model storage to infer.
- **Inference:**
  ○ The prediction application of the edge estimates the future environmental status based on the local data and the model transferred from the cloud platform.
  ○ This application can send meaningful results to the IoT interaction service as an input.
- **Service:**
  ○ This framework can provide service quickly based on the result at the edge, without network delay.

In this paper, this framework selects the correct predictive model that is generated by data from each space by looking at the highest correlation to select the most suitable model. In other words, this framework chooses the model with the highest correlation for a specific factor instead of choosing the model with the highest performance.

### 4.2. Test Process

We conducted a test to verify the validity of the framework proposed in this paper. The cloud platform builds LSTM network models for prediction. First, the cloud platform created prediction models based on the datasets collected from 29 spaces. Then, the cloud (or the edge) selects the best model among the candidate prediction models of the cloud, and the selected model is transmitted to the edge. We tested prediction models using the test dataset for the performance evaluation. This paper focuses on selecting the most appropriate model among already generated models, as opposed to improving the prediction accuracy by designing a new algorithm for individual cases. We considered a use case for predicting the future indoor status of PM10 and PM2.5. The predictive model can be used to predict the current indoor environment.

We evaluate the performance of the model that was delivered based on the proposed framework, rather than using a model delivered in the general cloud platform. In addition, this paper does not consider improvements of a specific algorithm to increase the absolute performance of the prediction.

### 4.3. Long-Short Term Memory Network

In this section, we briefly introduce the long-short term memory (LSTM) network used in our study. A recurrent neural network (RNN) is a network structure that can accept inputs and outputs regardless of sequence length. The RNN has neurons sending feedback signals to each other to remember past information, and various flexible RNN structures (RNNs) can be created as needed. The RNN structure can also classify and combine past and current information [46]. In addition, the RNN is a type of ANN designed to analyze the patterns of data sequences, such as text, handwriting, voice signals, IoT sensor data, or stock market data. However, if the distance between specific information and the point where the information is used is too far away, the gradient will decrease due to backpropagation, and the learning ability will decrease significantly. This is referred to as the vanishing gradient problem of RNNs. RNNs have been found to be unsuitable for long-term memory dependency because of this vanishing gradient problem, and the LSTM network was introduced to overcome these issues [47].

LSTM units are units of the RNN, and the RNN composed of LSTM units is called an LSTM network. LSTM is the structure that adds the cell state to the hidden state of the RNN. This model can be applied to handle time series data, such as sensor data. All RNNs have a chain form of repeating modules. In the case of standard RNNs, this module will have a very simple structure, like a tanh layer. LSTMs also have this chain structure, but the repeating module has a more complex structure. Instead of having a single neural network layer, they have four special units interacting in a special way. Generally, LSTM units are composed of a cell and three gates, such as an input gate, an output gate, and a forget gate. The cell remembers values during a specific time interval and the three gates control the flow of information. LSTM is well-suited to classify, process, and predict time series given time lags of unknown duration.

A standard RNN computes the hidden vector sequence $h=(h_{1},\;\ldots \;,\;h_{T})$ by iterating the following equations from t = 1 to T, where $x=\;(x_{1},\;\ldots \;,\;x_{T})$, and $y=\;(y_{1},\;\ldots \;,\;y_{T})$.

(1) $${\;h}_{t}=H\left(W_{xh}x_{t}+\;W_{hh}h_{t-1}+\;b_{n}\right)$$

(2) $$y_{t}=W_{xh}x_{t}+\;b_{y}$$

Here, the W terms denote weight matrices, b terms denote the bias vector, and H is a hidden layer function. H is an element-wise application of a sigmoid function. As we mentioned previously, the vanishing gradient problem occurs when gradients of the typical activation functions are multiplied many times. To avoid this problem, the LSTM algorithm was introduced. The LSTM cell contains the following components:

- f: Forget gate (a neural network with sigmoid)
- C: Candidate layer (a neural network with tanh)
- I: Input gate (a neural network with sigmoid)
- O: Output gate (a neural network with sigmoid)
- H: Hidden state (a vector)
- C: Memory state (a vector)

**Figure 5:**
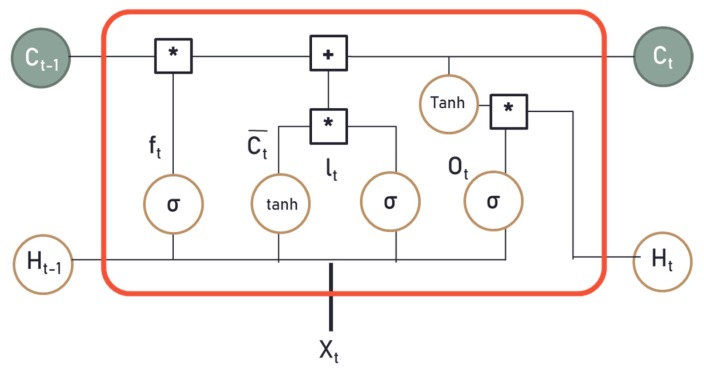
The diagram for an LSTM cell at time T.

Inputs into the LSTM cell at any step are the current input $X_{t}$, previous hidden state $H_{t-1}$, and previous memory state $C_{t-1}$. Figure 5 shows the diagram for LSTM at time step t with input $X_{t}$. The LSTM architecture used in this paper is given by the following equations, where σ is the logistic sigmoid function.

(3) $$I_{t}=\;\sigma {\left(W_{xi}x_{t}-W_{hi}h_{t-1}+b_{i}\right)}_{\;}\;\;\;\;\;\;\;\;$$

(4) $$f_{t}=\;\sigma {\left(W_{xf}x_{t}-W_{hf}h_{t-1}+b_{f}\right)}_{\;}\;\;\;\;\;\;\;\;$$

(5) $$O_{t}=\;\sigma (W_{xO}x_{t}-W_{hO}h_{t-1}+b_{O})$$

(6) $$\bar{C_{t}}=\;tanh{\left(W_{xo}x_{t}-W_{ho}h_{t-1}+b_{o}\right)}_{\;}.$$

(7) $$C_{t}=\;f_{t}*C_{t-1}+\;I_{t}*\;C_{t}{}_{\;}$$

(8) $$H_{t}=\;O_{t}*\tanh\left(C_{t}\right)$$

In this paper, the LSTM algorithm has been applied to develop a machine learning model that predicts the future value of indoor environmental features based on previous data. The first step is to create an instance of the sequential class, and the next step is to connect the layers. We designed the simple LSTM recurrent layer, which is comprised of memory units, and a fully connected layer that often follows LSTM layers and is used for outputting a prediction. 

We defined a simple LSTM network with 50 neurons in the first hidden layer and one neuron in the output layer for predicting values. We use the mean square error (MSE) loss function and the efficient Adam version of the stochastic gradient descent. The model will be fit for 200 training epochs with a batch size of 72 in this experiment.

### 4.4. Performance Index

To evaluate the performance, we adopted the root-mean-square error (RMSE) as a performance index. The RMSE was used to evaluate the absolute error. These indexes are calculated as follows:

(9) $$RMSE=\sqrt{\frac{1}{N}\overset{N}{\underset{i=1}{\sum }}\;{\left(O_{i}-P_{i}\right)}^{2}}.$$

Here, $O_{i}$ denotes the observed value, $P_{i}$ denotes the predicted value, and N denotes the number of evaluation samples. 

We tested whether the proposed method showed better prediction performance at the edge. The RMSE was used to evaluate the predicted value. We also changed the real predictive value to the categorical value as an index to profile the situation when it was interacting with the actual IoT. 

Then, the predicted value is compared with the category value of the actual value; the grade accuracy is also used as a performance measure. In the case of PM10, it is defined as “normal” if the value is less than 80 μg/m^3^ and “danger” if it is more than 80 μg/m^3^. For PM2.5, if the value is less than 35 μg/m^3^, it is defined as “normal,” and a higher value is defined as “danger” We use each model to predict the accuracy grade on the test dataset and then compare the predicted target to the actual answer.

## 5. Test Results

### 5.1. Experimental Data Collection

We collected indoor data from 29 spaces with the consent of users. Data were collected for about one year in Seoul, Korea. The data include indoor (PM10, PM2.5, humidity, temperature, CO_2_, noise, and VOCs) and outdoor (PM10, PM2.5, humidity, and temperature) environment sensor values.

Table 2 provides the range of data and error for each sensor node. There were many problems, such as turning off the sensor power or disconnecting the network for a certain period of time. Therefore, the least-contaminated data were selected for the test. The predictive models were trained with data collected from 15 March 2018 to 14 April 2018. When the candidate prediction model using training data from sample spaces were generated initially, cross-validation method was used for initial validation. Since we focused on finding the best model among candidate models in the cloud rather than increasing the absolute performance of each prediction model, 812 (29 models × 28 datasets) tests were performed repeatedly to evaluate the performance of models that could be used in 29 edge situations. Data sets collected from 15 April 2018 to 20 April 2018 from 29 spaces were used for testing the candidate models. We did not have any specific information about each station because of anonymization; we only know the values of the dataset. The collected data were used to create a predictive model to predict the future status of PM10 and PM2.5. PM10 and PM2.5 prediction model were generated using 29 indoor data set and 1 outdoor data set. Each individual data set consists of indoor data set collected independently in a specific space and common outdoor data set. Since the outdoor data could not be collected separately in each individual space, the same open data was used.

Each piece of data was given an ID and the characteristics of the data were analyzed first. The total number of collected data points for 29 locations from March 15 to April 20 is 1,501,620. Outdoor air conditions and basic environmental information were also collected from open data sources. The provided values were given in 1 min intervals. Table 3 show the summarized distribution information of data, which were used for training.

### 5.2. Trained Model Performance

To verify the framework, we first created candidate predictive models using training data from 29 spaces and tested them using test data. To generate model predicting the future status of PM10 and PM2.5, all features (Co2, VOCs, noise, indoor/outdoor temperature, and indoor/outdoor humidity) were used for training. Prediction models had an LSTM network structure. We developed the framework based on a Python 3 environment to create the model using the Scikit-learn, Pandas, NumPy, and Matplotlib packages. We also used Tensorflow and Keras frameworks for deep running development. To guarantee the objectivity of the performance, the network structure is designed and applied equally for each space’s model. The cloud platform generates an individual prediction model based on the training data of each space.

The first step of the test is to prepare the dataset for the LSTM. All features are normalized, then the dataset is transformed into a supervised learning problem. Both training and testing dataset needs to be reframed for the supervised learning problem as predicting the status of the future (t+a) given the indoor/outdoor air quality measurement at the prior time step. The target feature variables for the hour to be predicted (t+a) are used as comparison data for performance evaluation. Train and test dataset were split into input and output variables. All input of the predictive model is reshaped into the 3D format expected by LSTMs, namely [samples, timesteps, features] to be used.

We define the previous method and the proposed method as follows and use the RMSE as a basic evaluation method. The previous method is to provide the model with the lowest RMSE based on each test for a new edge. This previous method is a general cloud solution to deploy the predictive model for the new edge device. Alternatively, the proposed method of a new edge solution is to find the model that is generated by the specific space data that have a high correlation with the sample data from the local edge.

Table 4 shows the results of the first scenario based on the test data. Models with the best RMSE performance were selected for predicting the individual factors of each space. Additionally, we assumed that this selected model is the model that the cloud will transfer to the edge of all local edge spaces. The Id_003 model is considered to be the model with the lowest RMSE and the highest score accuracy; therefore, it was used as the model for the previous method.

### 5.3. Proposed Model Selection Results

In this section, we compare the results of the previous and proposed methods. In the case of PM2.5, the prediction model of ID_003, which has the smallest RMSE value, is selected as the best model for the previous method. For the performance evaluation, the future values of the other 28 spaces were predicted using this model and the average RMSE values were calculated.

To predict each individual factor of each space, the framework selects the best model with the highest correlation of the other 28 spaces and uses it for prediction at the edge. For performance evaluation, we conducted further experiments to determine the relationship between the correlation and RMSE of each dependent variable. To predict the future status of a specific space, we used the models using data from the other 28 spaces as prediction models. For each of the 29 spaces, 28 models were used for prediction. 29 × 28 predictions were performed and the RMSE and correlation values were calculated. Finally, we compared the RMSE average of the previous method with the RMSE average of the proposed method. RMSE is the main metric to evaluate the predicted value. Grade accuracy is one of the additional indicators that assess the performance for better understanding and is not objective compared to RMSE. We changed the real predictive value to the categorical value. In the case of PM2.5, the values from 0 to 30 were again encoded with the “normal” variable. A value of 30 or more is encoded as “danger”. In the case of PM10, the values from 0 to 80 were again encoded with the “normal” variable. A value of 80 or more is encoded as “danger”. In addition, accuracy was calculated by comparing the coded values of both predicted and original values again.

Table 5 shows the summarized results for three scenarios. The PM2.5 prediction results of the second scenario are described below. In the case of PM2.5, the average RMSE predicted for the 29 spaces in the previous method was 14.92, while the average RMSE predicted by the proposed method was 5.09. The average correlation between the PM2.5 data of a specific space (ID_003), which was the model used in the previous method, and the PM2.5 value of each space is very low (-0.10). When using the proposed method, the average PM2.5 data correlation value between the space of the dynamically selected model and the predicted space is high (0.78). In addition, the prediction values can be encoded into a binary class. The average grade accuracy of the encoded result of the previous method was 0.80, while the proposed method’s grade accuracy was 0.94. In other words, the grade accuracy of the proposed method is higher than that of the previous method. The prediction results for PM10 and PM2.5 considering other scenarios also show that the performance of the proposed method is better.

Table 6 shows the results of an additional test for the third scenario We generated the models using the data from each space and then tested the generated model using the test data collected in the other space. In other words, we tested using 28 models for 29 spaces. A high correlation group is the group of the top three spaces with the highest correlation with each space data. A low correlation group is the group of the top three spaces with the lowest correlation with each of the 29 spatial datasets. A middle correlation group is the group of other spaces, excluding the low and high correlation groups. The 29 spaces have different high, low and middle correlation groups. We used these groups to calculate the statistical characteristics according to the correlation value and validated each group for significant differences. Table 6 shows the average correlation, RMSE, and accuracy of 29 high/middle/low correlation groups. These results show that a higher correlation leads to better RMSE performance.

**Figure 6:**
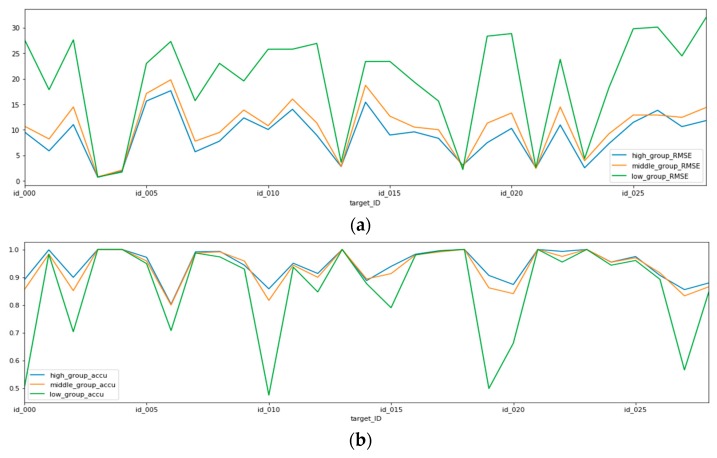
The average RMSE results of the high, middle, and low correlation groups for scenario three. (**a**) the RMSE averages in the low, middle, and high correlation groups for each space; (**b**) the average grade accuracies of each group in each space.

For the performance evaluation, we conducted an additional test to determine the relationship between the correlation and RMSE of each dependent variable. Figure 6 shows the test results of PM10 for scenario 1. The Figure 6a shows the RMSE averages in the low, middle, and high correlation groups for each space. There are little differences in the RMSE values among the three groups in some spaces, but the difference is clear in most spaces. In all cases, the higher the correlation, the better the RMSE performance. The Figure 6b shows the average grade accuracies of each group in each space. In all cases, the higher the correlation, the better the grade accuracy. Therefore, it is confirmed that the proposed model selection method helps improve the prediction performance on the edge side.

## 6. Conclusions

In this paper, we introduced a new design for a cloud-edge collaboration framework for IoT data analytics. The cloud generates candidate predictive models based on training data and selects the most appropriate model by finding the space that produces the most similar data pattern. The edge can use this selected model, which is trained by data from the selected space. The test results show that our proposed method has better performance than previous methods. To validate the performance, we considered a use case that predicts the future state of indoor PM10 and PM2.5. The outdoor air quality is similar in the near zone, but the effect on fine indoor dust concentration of each indoor is very different. For this reason, it is difficult to obtain good performance when using a general prediction model at all edges.

The proposed framework is specialized to protect private user data, while the edge actively selects and uses the most appropriate model from the cloud. Additionally, it can help analyze IoT heterogeneous sensor data at the edge side; the edge does not have enough power to make its own machine learning model with large data sets. The main advantages of the proposed framework are as follows. This framework has the potential to address concerns related to the response time requirement, bandwidth cost saving, and network dependency issues. It sends a minimum amount of data to the cloud, instead of sending all of the local data. It can also reduce privacy risks while processing local data at the edge without transferring private data to the cloud. Because the edge does not have enough processing power, it can also receive intelligence from the cloud and analyze IoT sensor data. In particular, the proposed framework is suitable for making inferences based on time-series sensor data that are affected by various factors, such as time and spatial information.

There are still some missing points we would like to address in the future. We considered only the magnitude of the correlation of the data as a way to find a better model. However, prediction results would be better if other complex factors, such as data trends, patterns, and abnormal signal generation of individual data, were considered together. We also have a plan to validate the proposed framework by applying various use cases.

## Figures and Tables

**Table 1:** Advantage vs. disadvantage of cloud and edge platforms in terms of data analysis service.

	Advantage	Disadvantage
**Cloud**	- Powerful processing resources - Computational efficiency - Easy to use - Cost savings - Efficient management - Massive storage - Wide-area coverage	- Privacy concerns - High bandwidth costs - Network connection dependent - Latency problems - Service provider dependent
**Edge**	- Real-time data handling - User-centric process - High mobility - Privacy protection - Network connection independence - High QoS	- Low processing power - Insufficient storage - Different specification - Only local-area coverage

**Table 2:** Specifications of six sensor modules used for air quality measurement.

Factor	Unit	Data Range	Error Range
CO₂	ppm	0–10,000 ppm	±20 ppm ±3%
VOCs	ppb	125–3500 ppb	±10–30% (123–3500)
PM10	μg/m^3^	0–500	±10%
PM2.5	μg/m^3^	0–500	±10%
Temperature	°C	−40–125	±0.3 °C (20–40 °C) ±1.0 °C (0–70 °C)
Humidity	%	0–100	±2.0% (20–80%) ±4.0% (0–100%)
Noise	dB	30–90	±5 dB

**Table 3:** Data distribution information of indoor and outdoor environmental factors.

	Indoor							Outdoor			
	CO₂	VOCs	Noise	PM10	PM2.5	Temperature	Humidity	PM10	PM2.5	Temperature	Humidity
**mean**	735.89	322.19	55.64	41.12	29.34	22.14	36.70	53.92	31.89	10.28	59.66
**std**	458.15	266.56	12.25	36.23	24.79	2.51	8.93	42.81	28.31	5.41	22.02
**min**	1.95	125.00	32.00	5.00	3.00	11.09	10.80	3.00	1.00	0.10	12.31
**0.25%**	491.56	171.41	44.09	15.78	11.27	20.75	31.00	22.00	10.00	6.30	42.50
**0.50%**	617.64	247.06	58.00	23.30	16.77	22.50	37.00	50.00	25.00	9.80	58.42
**0.75%**	866.24	360.90	66.09	60.13	44.00	23.75	43.00	75.00	44.00	14.60	78.76
**max**	5000.0	2000.0	81.27	500.0	352.7	32.31	74.20	380.00	138.00	23.70	97.90

**Table 4:** Test data results using each model with the first scenario.

	PM10		PM2.5	
ID	Grade Accuracy	RMSE	Grade Accuracy	RMSE
id_000	0.937	9.147	0.888	6.626
id_001	0.993	5.587	0.988	3.649
id_002	0.967	10.103	0.927	6.172
**id_003**	**1.000**	**0.818**	**1.000**	**0.567**
id_004	1.000	2.267	0.995	1.973
id_005	0.974	9.801	0.965	6.491
id_006	0.934	12.676	0.902	8.620
id_007	0.995	5.760	0.991	3.541
id_008	0.988	9.129	0.967	5.829
id_009	0.953	12.793	0.829	9.802
id_010	0.895	9.546	0.918	6.993
id_011	0.965	12.342	0.953	8.461
id_012	0.972	9.467	0.895	6.033
id_013	1.000	2.100	0.993	1.733
id_014	0.974	8.168	0.977	5.851
id_015	0.974	9.726	0.906	6.846
id_016	0.986	8.465	0.958	6.602
id_017	0.953	9.142	0.951	6.579
id_018	1.000	2.389	1.000	1.825
id_019	0.965	7.658	0.930	5.259
id_020	0.965	10.935	0.871	6.947
id_021	1.000	2.526	1.000	1.893
id_022	0.956	15.950	0.831	10.282
id_023	0.995	3.651	0.991	2.492
id_024	0.981	8.558	0.899	6.708
id_025	0.974	11.595	0.946	7.740
id_026	0.958	8.216	0.951	6.257
id_027	0.911	10.295	0.881	7.781
id_028	0.981	6.788	0.918	5.046

**Table 5:** Prediction results of three scenarios.

		Scenario 1		Scenario 2		Scenario 3	
		PM10	PM2.5	PM10	PM2.5	PM10	PM2.5
**Previous method**	Average RMSE	27.11	18.93	**23.26**	**14.92**	10.15	7.20
	Average grade accuracy	0.80	0.73	**0.87**	**0.80**	70.94	0.78
**Proposed method**	Average RMSE	8.60	6.01	**7.45**	**5.09**	23.42	16.65
	Average grade accuracy	0.94	0.94	**0.96**	**0.94**	0.82	0.92

**Table 6:** PM10 and PM2.5 prediction results based on the correlation ranking.

			Scenario1		Scenario 2		Scenario 3	
		Average Correlation	Average RMSE	Average Grade Accuracy	Average RMSE	Average Accuracy	Average RMSE	Average Grade Accuracy
**PM 10**	Low correlation group	**0.06**	19.77	0.86	18.46	0.89	19.52	0.87
	Middle correlation group	**0.40**	10.61	0.93	9.13	0.94	12.68	0.92
	High correlation group	**0.75**	**8.92**	**0.94**	**7.53**	**0.95**	**10.57**	**0.94**
**PM 2.5**	Low correlation group	**0.06**	14.39	0.79	12.27	0.83	13.36	0.82
	Middle correlation group	**0.39**	7.50	0.92	6.50	0.92	9.02	0.90
	High correlation group	**0.74**	**6.18**	**0.93**	**5.23**	**0.94**	**7.42**	**0.92**
